# Impact of III-Nitride/Si Interface Preconditioning on Breakdown Voltage in GaN-on-Silicon HEMT

**DOI:** 10.3390/mi12111284

**Published:** 2021-10-21

**Authors:** Abdelkrim Khediri, Abbasia Talbi, Abdelatif Jaouad, Hassan Maher, Ali Soltani

**Affiliations:** 1 Laboratoire de Microélectronique Appliquée, Université Djillali Liabès de Sidi Bel Abbès, Sidi Bel Abbès 22000, Algeria; talbi_a02@yahoo.fr; 2 Plateforme Technologique de Micro-Fabrication, Centre de Développement des Technologies Avancées, Algiers 16081, Algeria; 3 Laboratoire Nanotechnologies Nanosystèmes (LN2)—CNRS, Université de Sherbrooke, 3000 Boulevard de l’Université, Sherbrooke, QC J1K 0A5, Canada; abdelatif.jaouad@usherbrooke.ca (A.J.); hassan.maher@usherbrooke.ca (H.M.); ali.soltani@usherbrooke.ca (A.S.); 4 IEMN (Institut d’Electronique de Microélectronique et Nanotechnologie), CNRS-UMR-8520, University of Lille, 59655 Villeneuve d’Ascq, France

**Keywords:** GaN, HEMT, parasitic current path, high voltage breakdown

## Abstract

In this paper, an AIGaN/GaN metal-oxide-semiconductor high-electron-mobility transistor (MOS-HEMT) device is realized. The device shows normal ON characteristics with a maximum current of 570 mA/mm at a gate-to-source voltage of 3 V, an on-state resistance of 7.3 Ω·mm and breakdown voltage of 500 V. The device has been modeled using numerical simulations to reproduce output and transfer characteristics. We identify, via experimental results and TCAD simulations, the main physical mechanisms responsible for the premature breakdown. The contribution of the AlN/Silicon substrate interface to the premature off-state breakdown is pointed out. Vertical leakage in lateral GaN devices significantly contributes to the off-state breakdown at high blocking voltages. The parasitic current path leads to premature breakdown before the appearance of avalanche or dielectric breakdown. A comparative study between a MOS-HEMT GaN on a silicon substrate with and without a SiNx interlayer at the AlN/Silicon substrate interface is presented here. We show that it is possible to increase the breakdown voltages of the fabricated transistors on a silicon substrate using SiNx interlayer.

## 1. Introduction

AlGaN/GaN high electron mobility transistors (HEMT) have attracted an increasing interest for their high efficiency power electronics benefit from the electron transport properties and the high critical electrical field of this wide band gap material [1]. GaN-on-Si is highly attractive as a high performance technology with low cost. However, the high reactivity of silicon with the different compounds frequently used for the growth of nitrides (Ga, Al, and N precursors) makes the substrate preparation and the nucleation more delicate than on a substrate like SiC [2]. Furthermore, GaN-on-Si suffers from a risk of high dislocation density or crack generation due to the tensile stress induced by the large lattice mismatch (17%) and thermal expansion coefficient difference between GaN and Si. The parasitic diffusion of dopant species into the silicon substrate [3,4,5], as well as degraded crystal quality at the AlN/Si interface, have been reported as possible origins of leakage paths limiting the reliability of such structures at high voltages, which leads to premature breakdown of the transistor.

The control of the electrical behavior of HEMT structures is still challenging. Both sufficient crystal quality and electrical resistivity regarding the AlN/Silicon interface are required for achievement of high breakdown voltage. The use of an interlayer between the AlN nucleation layer and silicon substrate is a promising alternative to reduce the interface states and eliminate a conductive path. Cubic Silicon Carbide (3C-SiC) was proposed as a template with a reduced lattice mismatch with GaN as well as a reduced thermo-elastic strain after GaN based structure regrowth [6].

Previous approaches for boosting the breakdown have focused on improving the growth conditions of the buffer layer (particularly strain relief) in order to reduce the oxygen impurities and threading dislocations identified as responsible for the leakage paths [7]. Other groups worked on increasing the buffer thickness for boosting the breakdown [8].

In the literature, there are plenty of articles concerning the simulation of GaN HEMTs by exploiting TCAD simulators [9,10,11,12]. Most of these studies have been focused on the most active area of the transistor. Few studies investigate the influence of the substrate and nucleation layers on breakdown. G. Longobardi et al. [13] proposes a TCAD approach for simulating the non-ideality of the AlN nucleation layer and AlN/Si interface. Li, X. et al. [14] investigate carrier transport through AlN from a different doping nature of Si(111) substrates. In this work, we elaborate on a normal ON AIGaN/GaN metal-oxide-semiconductor high-electron-mobility transistor (MOS-HEMT) device on-Si for switching electronics and we investigate, using ATLAS-SILVACO software, simulations of the premature breakdown in the device. We show that eliminating leakage at AlN/Si interface makes it possible to increase the breakdown voltages of the fabricated transistors based on a GaN-on-Silicon substrate. Our approach is to use a SiNx layer for cost effective leakage reduction.

## 2. Materials and Methods

### 2.1. Current-Voltage Characteristics

**Figure 1:**
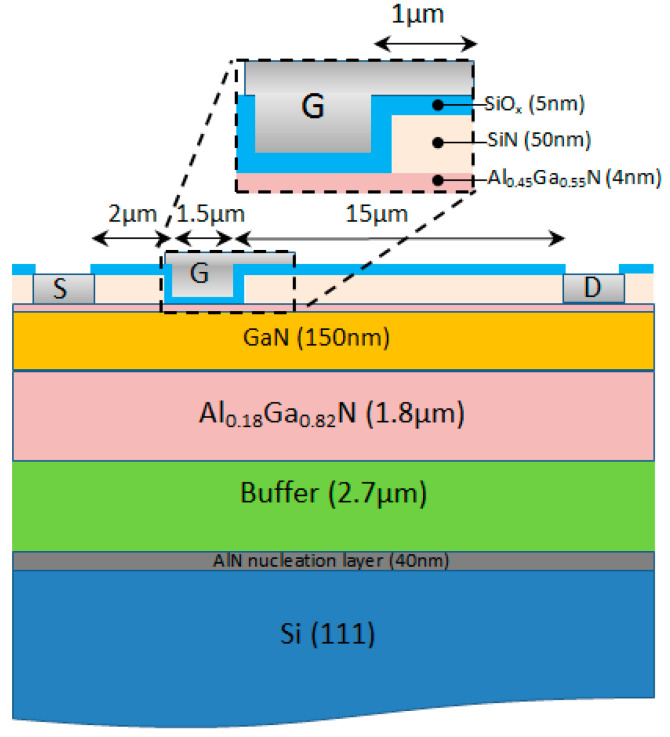
Metal-oxide-semiconductor high-electron-mobility transistor (MOS-HEMT) cross section.

Normal ON AIGaN/GaN MOS-HEMT devices were realized on commercial HEMT substrate. The composition of the transistor includes: a highly resistive Si substrate, an AlGaN buffer with a back barrier grown on 40 nm of an AlN nucleation layer, a 150 nm GaN non-intentionally doped channel, a 4 nm Al_0.45_Ga_0.55_N barrier and 5 nm SiO_2_ gate oxide. The transistors have the following dimensions: a gate length of 1.5 µm, 1 µm gamma field plate, a source-drain distance (L_GS_) of 2 µm, and 15 µm gate-drain (L_GD_) distance. The epitaxial structure used for this study is illustrated in Figure 1.

The device was simulated by using ATLAS-SILVACO software. Poisson’s and continuity equations, including ionized deep-level terms for electrons and holes and a drift-diffusion model, are used to solve transport equations. The simulations include Schockley–Read–Hall, Auger recombination and carrier-dependent lifetimes. The electron saturation velocity and mobility model are taken according to Farahmand’s theory [15]. The composition and temperature-dependent low field model defined by:

µ_n_ (T,N) = m1·(T⁄300)^b^ + [(m1 − m2)·(T⁄300)^d^]/(1 + [N/(Ncr∙(T⁄300)^g^)]^(a∙(T⁄300)^^)^^^E^) (1)

For the high field mobility, nitride specific field dependent mobility model was used [15] as described by the following equation:

µ_n_ = (µ_n_ (T,N) + Vsat∙(E^(N1−1)^/Ecn^N1^))/(1 + an∙(E/Ecn)^N2^ + (E/Ecn)^N1^) (2)

The coefficients were set in agreement with Monte Carlo calculations.

Polarization charges of 10^13^ cm^2^ are set at the barrier/channel interface, and surface states are included through a 2.3 × 10^12^ cm^−2^ fixed donor trap density, uniformly distributed on the AlGaN/Oxide interface.

In GaN power devices, carbon (C) is widely adopted as compensation doping to suppress the unintentional conductivity in the GaN buffer and transition layers underlying the MOS-HEMT channel. Additionally, it is used to avoid premature breakdown related to source-to-drain punch-through. The C doping is modeled by means of acceptor and donor type traps associated, respectively, with the C_N_ and C_Ga_ states [16]. For high-resistivity silicon, we use a p-type doping substrate with a uniformly distributed concentration of 1.47 × 10^12^ cm^−3^. Table 1 lists the main physical parameters included in the simulations.

### 2.2. Breakdown Characteristics

We considered bulk traps uniformly distributed in all GaN-buffer layers, with a concentration of 3.37 × 10^15^ cm^−3^ and 7.5 × 10^15^ cm^−3^ for the donor and acceptor traps, respectively, corresponding to E2 and H1 carbon-related defects [16]. In addition, the trap level E4 is used in the simulation, consistent with a deep double donor located around at 0.74 eV below the conduction band as described by Ghazi et al. [16,17], which is attributed to native point defects in the GaN films. Electron and hole capture cross sections of 1 × 10^−15^ cm^2^ are used for all trap levels, which are consistent with other reported measurements [18]. A positive polarization charge, equal to 5 × 10^13^ cm^−2^, is used at the AlN/Si interface and the consequent electron accumulation is generated at the same interface. To model the transport mechanism, trap assisted tunneling (TAT) [19] through AlN and discreet traps were located within the AlN layer. The TAT model is used with the acceptor traps, uniformly distributed in the AlN layers, with a density of 5 × 10^16^ cm^−3^ and being defined at level 0.6 eV below the conduction band based on the works of G. Longobardi et al. [13]. We used the impact ionization as high field carrier generation to reproduce the experimental breakdown voltage [20].

In order to simulate the off-state breakdown voltage (V_BD_), the device was first biased under pinch-off. Here, gate-to-source voltage (V_GS_) equals −8 V, which is lower than the threshold voltage V_T_. Then, the drain voltage was increased until V_BD_ was reached. We define V_BD_ as the voltage value when the compliance current fixed in experimental measurements (1 mA/mm) is reached.

## 3. Results

**Figure 2:**
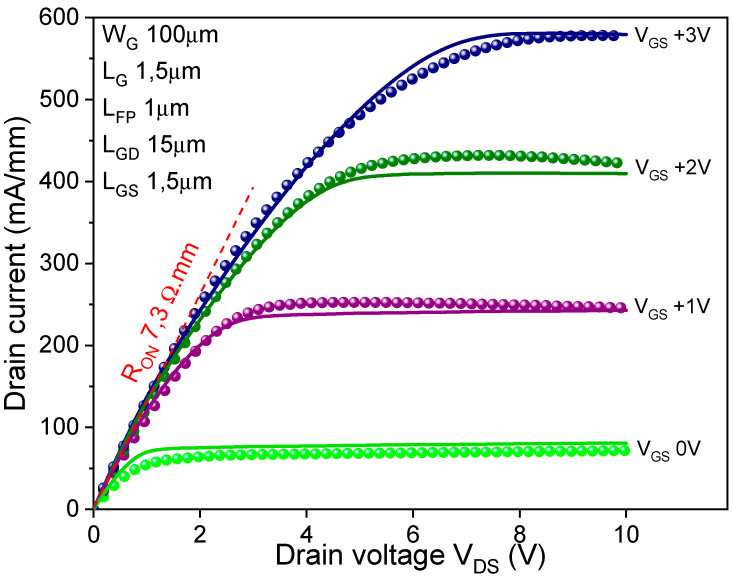
Experimental (doted) and simulated (line) output characteristics of the device for V_GS_ from 0 to +3 V.

**Figure 3:**
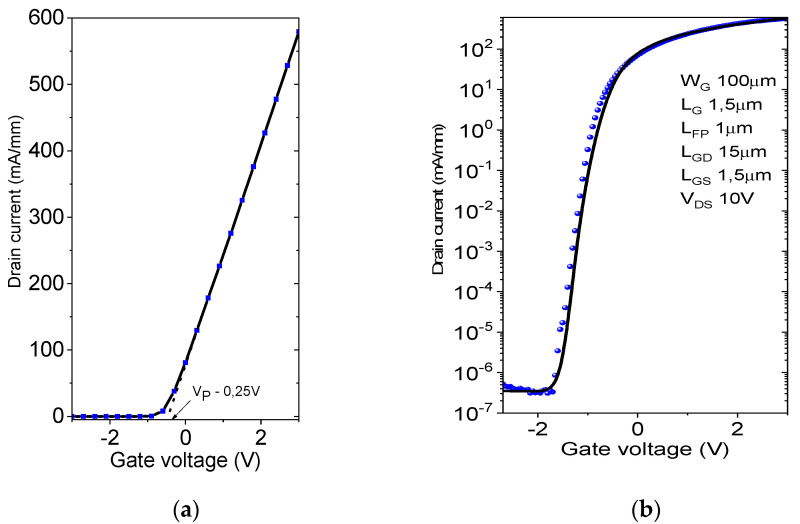
Experimental (doted) and simulated (line) transfer characteristic (I_DS_-V_GS_) of AlGaN/GaN MOS-HEMT device (**a**) in linear and (**b**) logarithm scale.

First of all, DC measurements were performed on the devices before measuring the breakdown voltage. Simulations were then calibrated against experimental transfer and output IV curves. The maximum I_DS_ was more than 570 mA/mm at V_GS_ value of 3 V. The devices exhibited a pinch-off voltage of −0.25 V with a leakage current below 10^10^ A/mm. Drain current density (I_DS_) versus drain-to-source voltage (V_DS_) characteristics at a gate-to-source voltage (V_GS_) from 0 to +3 V of “fresh” device are shown in Figure 2. As shown in Figure 2, the device demonstrates an on-state resistance (R_ON_) of 7.3 Ω·mm. We notice that we did not observe the kink effect in the elaborated device in the measured range (V_DS_ 0–10 V), meaning that the electronic surface states near the top active areas are not predominant in our MOS-HEMT. The outcomes of the experimental and simulated transfer curves are shown in Figure 3. Applying the parameters specified above, a good fit is obtained.

**Figure 4:**
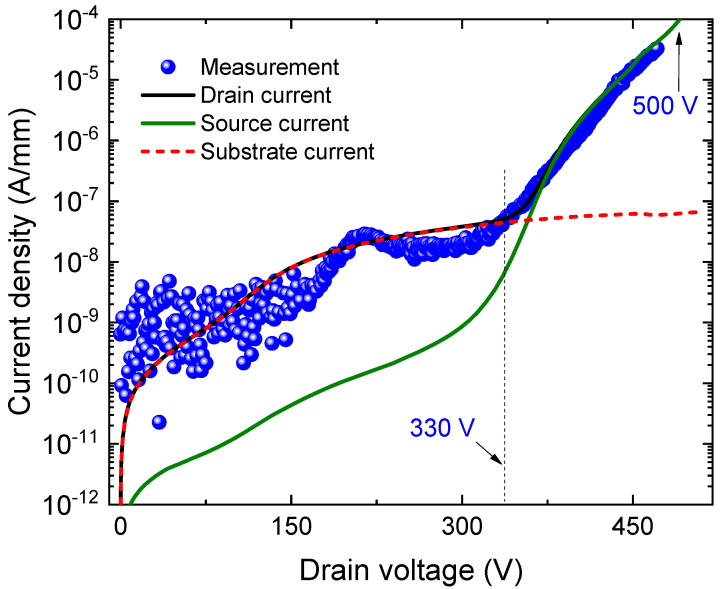
Experimental (doted) and simulated (line) AlGaN/GaN MOS-HEMT breakdown.

Three terminals measurements under pinch off conditions (V_GS_ = −8 V, V_substrate_ = 0 V) were carryout. The breakdown voltage was about 500 V, as shown in Figure 4. At a low drain voltage below 330 V, the substrate current dominated the drain current. It is verified that the gate current was not the trigger of the device breakdown. The rapid increase of the gate current was not observed at the breakdown. On the other hand, at high drain voltage over 330 V, the drain current increases rapidly and simultaneously with the source current. The substrate current remains at same level. The first stage of breakdown occurred with the increase of the drain and source currents at 400 V. Therefore, the source-to-drain leakage current (I_DS_) is considered to be the breakdown trigger.

## 4. Discussion

**Figure 5:**
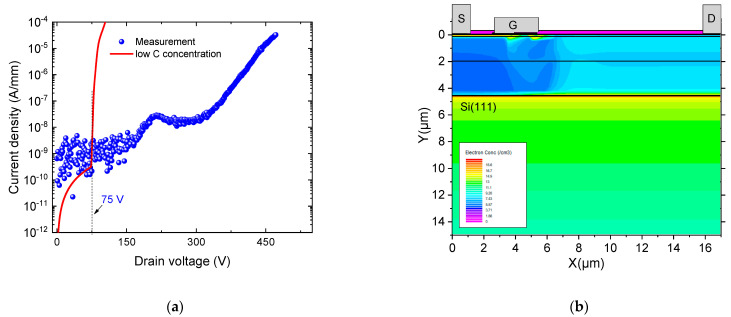
MOS-HEMT breakdown characteristics (**a**) Experimental (doted) and simulated (line) with low carbon concentration. Acceptor trap concentration of and 7.5 × 10^14^ cm^−3^ and donor trap concentration of 3.37 × 10^14^ cm^−3^ were considered. (**b**) MOS-HEMT structure cross section of electrons concentration at 100V showing more leakage across the buffer.

As the carbon was not intentionally introduced in these samples, the concentration of the acceptor trap along with a compensating donor trap was chosen at a low level according to U. Honda et al. [16]. The concentration values were varied to fit the experimental results with a dominating C-related acceptor. An agreement with the experimental data, as shown in Figure 4, was found for concentrations indicated in Table 1. With an even lower carbon traps concentration, more leakage across the buffer is observed and the breakdown takes place earlier, at 100 V, through the buffer, as shown in Figure 5. In our MOS-HEMT sample, the leakage path determining breakdown happens between the source and the drain contacts, with electrons injection from the source to the AlN/Si interface and then back towards the drain, as will be discussed in next section.

### 4.1. Breakdown Origin

**Figure 6:**
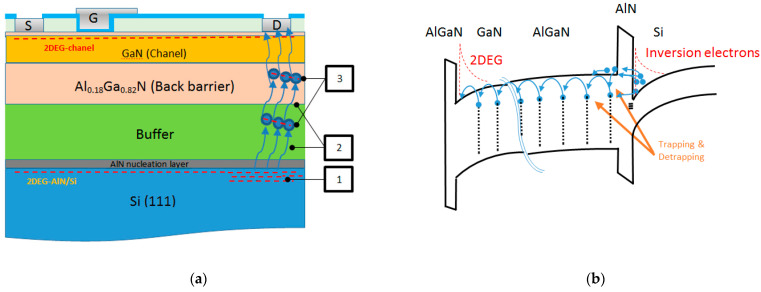
Physical mechanisms responsible for the vertical leakage: (**a**) Leakage current from AlN/Si interface through the AlN and buffer layer to the drain electrode (1) 2DEG at the AIN/Si interface constitutes the main source of electrons at high leakage currents. (2) Electrons are injected into the transition layers. (3) The current is limited by trapping of electron into acceptor states (SCLC). (**b**) The mechanisms of electron injection across the AIN barrier.

**Figure 7:**
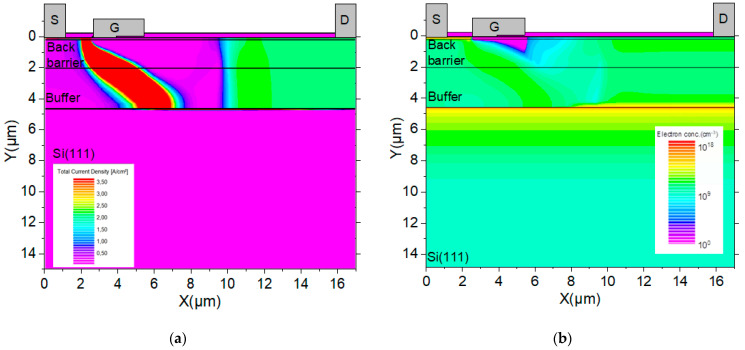
MOS-HEMT structure cross section of (**a**) current density and (**b**) electrons concentration at V_BD_ showing the current path from the drain to the source electrode and the 2DEG at AlN/Si interface. The bias voltage at the drain contributes to the accumulation of electrons drain side.

Dislocations threading through the pinched two dimensional electron gas (2DEG) channel can trap charges and act as vertical charge transport sites that can result in device leakage (for drain voltage below 330 V). At drain voltages close to the breakdown value, electrons become highly energetic carriers (hot electrons) and are injected from the source into the substrate. Due to the large voltage difference between the drain contact and the substrate, significant electron injection can take place from the substrate into the buffer (via thermionic emission, tunneling and hopping). The final collection to the drain terminal is illustrated in Figure 6. It is worth noticing that the impact ionization in silicon is not the main source of electrons. Without the source of electron-holes created by impact ionization, the substrate current does not increase rapidly at high voltages and remains at very low levels. So, the limiting factor to breakdown still the lateral existing electronic channel at the AlN/Si interface. The transport and physical mechanisms responsible of high vertical leakage are treated in details by Longobardi et al. [13] and Meneghesso et al. [21]. Here, the leakage current path limiting breakdown is the lateral AlN/Si conductive interface. From Figure 7 we can see the current path and the electrons’ concentration along the MOS-HEMT structure at the breakdown voltage (V_BD_). It is obvious that the current path through the existing electronic channel at the AlN/Si interface promotes premature breakdown. Indeed, the interface between Si and AlN is expected to be highly defective due to the large lattice and thermal mismatch between these materials. Furthermore, the AlN films are typically strained in tension (>1 GPa). Hence, the piezoelectric polarization is added to the spontaneous polarization. Since AlN on Si is almost always Al-polar, a positive polarization charge would be manifest at this interface. This will increase the interfacial sheet charge and consequently the inversion layer of electrons is formed at this interface for p-type substrate. A potential well that confines carriers closer to the interface forms a 2DEG, as can be depicted in Figure 6 and Figure 7. So, a parasitic current path occurs at the AlN/Si interface that limits the breakdown voltage.

### 4.2. Breakdown Improvement

Silicon substrate-removal and a layer-transfer process were proposed to enhance the breakdown voltage (V_BD_) for HEMT GaN-on-Si [22]. After Si removal, we measured a V_BD_ enhancement of devices with a gate-drain (L_GD_) distance of 15 μm and a V_BD_ > 1100 V compared with ~300 V for devices with a Si substrate. Improvement in V_BD_ is also observed experimentally when C-doping concentration was increased [23]. Furthermore, increasing the acceptor trap concentration while having a constant donor trap concentration also results in an improved V_BD_ (not shown here). The relative concentration of acceptor and donor traps controls the breakdown voltage in these devices. Another approach to enhance the breakdown voltage is to insert interlayer with the objective of elimination, 2DEG at the AlN/Si and/or interface state improvement.

Practically, the Si/III-nitride interface alone can typically be composed of SiNx layers due to the diffusion of nitrogen from the III-N films or the intentional nitridation of the Si surface [24]. However, the formation of SiNx layers is very much dependent of growth conditions. These SiNx layers are typically amorphous, thin (from few mono-atomic layers to few nanometers), and/or discontinuous and may act as diffusion barriers for the movement of other species to the Si surface. The effect of the SiNx layer on the III-N films in the literature is controversial. It has been reported that amorphous SiNx is not desirable because of the formation of deep-level generation centers in the AlN layer [25]. However, it has been demonstrated that such nitridation also reduces the dislocation density by using SiNx inclusions as in situ masking layers [24]. Here, we do not discuss the effect of nitridation on the above AlN layer. This will be discussed in future work. In the following paragraph, we try to simulate the effect of Silicon nitridation on V_BD_.

Taking the same structure and parameters described above, the MOS-HEMT is simulated by introducing 3 nm of SiNx. This SiNx layer is supposed to not affect the trap concentration and/or dynamic effects in the upper layers of the device. An easy-to-implement TCAD approach for simulating the non-ideality of the SiNx layer leakage current and tunneling is introduced through the layer.

**Figure 8:**
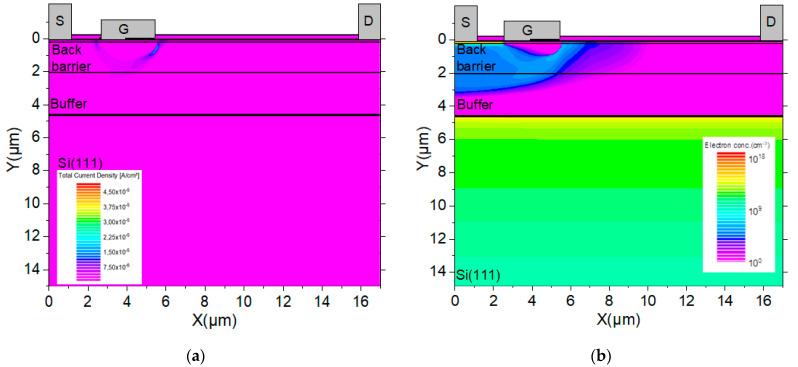
MOS-HEMT structure cross section of (**a**) current density and (**b**) electrons concentration at 500 V showing no current path through the 2DEG at AlN/Si interface. A small leakage current 10^−14^ A/mm through the buffer is denoted.

As we can see from Figure 8, at 500 V drain bias voltage, electrons are accumulated at the SiNx/Si interface and no breakdown is observed. However, we note a small current leakage about 10–14 A/mm through the buffer. As the voltage on the drain side becomes larger, an electron tunnel emerges at a strengthened vertical electric field. These tunneling effects intensify with the increase of the electric field amplitude. The tunnel effect is largely related to the band difference as well as the thickness of the SiNx.

**Figure 9:**
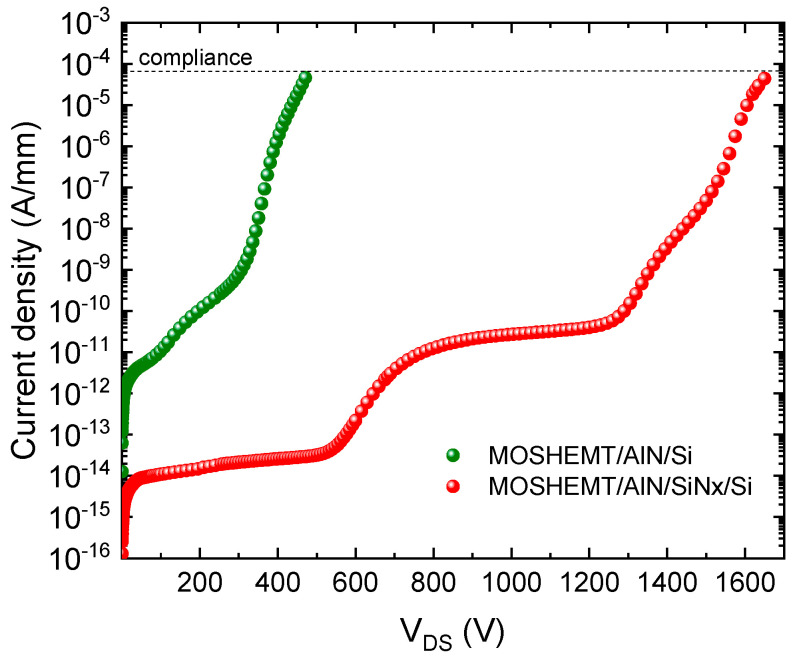
Drain current Vs drain voltage of MOS-HEMT with (V_BD_ = 1500 V) and without SiNx interlayer (V_BD_ = 500 V).

The breakdown voltage in the off state accrues around 1500 V, which is three times greater than the device without 3 nm SiNx layer. Drain current versus drain voltage for the MOS-HEMT structure with and without the SiNx interlayer is represented in Figure 9.

From the simulations, it is clear that if the crystalline quality of deposited interlayers (SiNx) and leakage current to the interface with the Si(111) substrate are improved, the breakdown voltage can be significantly enhanced and the GaN theoretical breakdown value can be reached. Here, the current path at the AlN/Si interface was not eliminated with the interlayer, and a leaking SiNx layer is used in the simulation.

As the voltage on the drain side becomes larger, an electron tunnel emerges at a strengthened vertical electric field; these tunneling effects intensify with the increase of the electric field amplitude. The tunnel effect is largely related to the band difference as well as the thickness of the SiNx. The breakdown voltage in the off state accrues around 1500 V, which is three times greater than the device without a 3 nm SiNx layer. Drain current versus drain voltage for the MOS-HEMT structure with and without the SiNx interlayer is represented in Figure 9.

Finally, it is notable that for high voltage in switched-mode operation, the thermal conduction and heat dissipation is an important parameter. The use of a good thermal conductive layer such as SiC potentially offers the excellent device characteristics required for high-power device applications. For high frequency applications, it is essential to use ultra-high resistivity wafers to eliminate the substrate loss. It was widely believed that the RF parasitic loss was due to the low resistivity of the Si substrate. Therefore, to minimize the substrate-dependent attenuation of GaN-HEMT on Si, it is required to suppress or at least isolate the sheet mobile electrons underneath the AlN buffer. In this work, we simulate the introduction of thin SiNx at the AlN/Si interface which can act as a loss suppression layer but we focused our study on the effect of the substrate on the breakdown voltage of HEMT transistors.

## 5. Conclusions

In this study, we have analyzed the off-state, three-terminal, breakdown of fabricated AlGaN/GaN HEMTs for power switching applications. The fabricated MOS-HEMT on Si exhibited a breakdown voltage of 500 V, an on-resistance of 7.3 Ω·mm, and a maximum drain current of more than 570 mA/mm at a gate-to-source voltage of 3 V. The electrical behaviors in normal and off-state breakdown conditions were successfully captured by our simulations and the 2DEG channel at AlN/Si has been taken into account for breakdown mechanism simulation. The premature V_BD_ of the sample was explained by the simulations as a result of the electron’s injection from the source to the AlN/Si interface and then to the drain through a parasitic current path at the AlN/Si interface. Indeed, as the AlN/Si interface is more conductive than the III-nitride layers with the presence of 2DEG, the breakdown at this interface is the most likely mechanism. By introducing the SiN interlayer, we found that the breakdown voltage values can significantly be improved to 1500 V. From these results, it is prominent that the structural and electrical characteristics of the AlN/Si interface greatly influence the breakdown characteristics for GaN-on-Si HEMT.

## Figures and Tables

**Table 1:** Geometrical and model parameters of the device used in the simulations.

Physical Mechanism	Model	GaN	AlGaN(Back Barrier + Buffer)	AlN
Dimensions	-	150 nm	1.8 + 2.7 µm	40 nm
Low field mobility	Farahmand’s Law	m1 = 295 (cm^2^·V^−1^s^−1^)	m1 = 132 (cm^2^·V^−1^s^−1^)	m1 = 297 (cm^2^·V^−1^s^−1^)
		m2 = 1460 (cm^2^·V^−1^s^−1^)	m2 = 306 (cm^2^·V^−1^s^−1^)	m2 = 683 (cm^2^·V^−1^s^−1^)
		a = 0.66	a = 0.29	a = 1.16
		b = −1.02	b = −1.33	b = −1.82
		d = −3.43	d = −1.75	d = −3.43
		g = 3.78	g = 6.02	g = 3.78
		E = 0.86	E = 0.41	E = 0.86
		Ncr = 10^17^	Ncr = 10^17^	Ncr = 10^17^
High field mobility	Farahmand’s Law	Vsat = 1.9 × 10^7^ (cm/S)	Vsat = 1.27 × 10^7^ (cm/S)	Vsat = 2.167 × 10^7^ cm/S)
		Ecn = 220 (kV/cm)	Ecn = 365 (kV/cm)	Ecn = 447 (kV/cm)
		N1 = 7.2044	N1 = 5.3193	N1 = 17.368
		N2 = 0.7857	N2 = 1.0396	N2 = 0.8554
		an = 6.1673	an = 3.2332	an = 8.7253
Unintentional Doping	Trap Energy level (eV)/Density (cm^−^^3^)	E2 (Ec − 0.4)/1 × 10^15^	E2 (Ec − 0.4)/1 × 10^15^	
		E4 (Ec − 0.74)/3.37 × 10^15^	E4 (Ec − 0.74)/3.37 × 10^15^	E1 (Ec − 0.6)/5 × 10^16^
		H1 (Ev + 0.86)/7.5 × 10^15^	H1 (Ev + 0.86)/7.5 × 10^15^	
